# The Environmental Polycyclic Aromatic Hydrocarbon (PAH) Benzo[a]pyrene (BP) Alters SARS-CoV-2 Pathogenesis in a Mouse Model of Disease

**DOI:** 10.3390/v18080823

**Published:** 2026-07-26

**Authors:** Jennifer L. Spencer Clinton, Freedom M. Green, Emma E. Crotty, Yike Jiang, Weiwu Jiang, Sarah Strobel, Fong W. Lam, Bhagavatula Moorthy, Shannon E. Ronca

**Affiliations:** 1 Department of Pediatrics, Division of Tropical Medicine, Baylor College of Medicine and Texas Children’s Hospital, Houston, TX 77030, USA; 2 William T Shearer Center for Human Immunobiology, Texas Children’s Hospital, Houston, TX 77030, USA; 3 Department of Molecular Virology and Microbiology, Baylor College of Medicine, Houston, TX 77030, USA; 4 Department of Pediatrics, Division of Critical Care, Baylor College of Medicine and Texas Children’s Hospital, Houston, TX 77030, USA; 5 Department of Pediatrics, Division of Neonatal-Perinatal Medicine, Baylor College of Medicine and Texas Children’s Hospital, Houston, TX 77030, USA

**Keywords:** SARS-CoV-2, pollutant, environmental exposure, murine models

## Abstract

Since emerging in late 2019, SARS-CoV-2 has caused over 7 million deaths globally and remains a public health concern. Understanding SARS-CoV-2 pathogenesis is vital, especially as factors like environmental exposures are still poorly understood. Polycyclic aromatic hydrocarbons (PAHs), like benzo[a]pyrene (BP), found in pollutants like cigarette smoke, diesel exhaust, and charcoal-broiled steaks, are known to injure the lungs. We aimed to evaluate if BP exacerbates SARS-CoV-2 pathogenesis in a mouse model of disease. One day following intranasal administration of BP (20 mg/kg) or vehicle control, we infected male and female K18-hACE2 mice with ancestral SARS-CoV-2 and assessed lung viral load, weight change, clinical scores, immune cell recruitment, and survival in the presence and absence of BP exposure. We found that BP-exposed mice had decreased survival compared to mock-exposed mice. Additionally, BP did not alter innate or adaptive immune cell populations in the lungs of SARS-CoV-2-infected mice. These findings suggest that PAH exposure exacerbates severe COVID-19 outcomes by unknown mechanisms, highlighting the need to further explore environmental impacts on SARS-CoV-2 infection.

## 1. Introduction

SARS-CoV-2, the causative agent of COVID-19, remains a serious concern to human health. COVID-19 has caused >7 million deaths worldwide, with an estimated 778 million total infections [1]. Although scientists rapidly developed safe and effective vaccines, SARS-CoV-2 continues to cause disease throughout the globe. Even among vaccinated populations, the severity of symptoms ranges from mild to severe, and the factors that influence disease outcomes are still not fully understood. Recent evidence supports that environmental pollutants and poor air quality are some of the co-factors driving differences in COVID-19 lethality.

Environmental pollutants include polycyclic aromatic hydrocarbons (PAHs), organic pollutants generated from combustion of fossil fuels, biomass, tobacco, or other organic materials. These compounds are pervasive in the environment and enter the human body through inhalation, ingestion, or dermal contact. PAHs are well known to have carcinogenic, mutagenic, and immunomodulatory properties [2], and have been linked to various chronic health conditions including cancers, cardiovascular disease, reproductive dysfunction, and respiratory disorders [3,4]. Their persistence in the environment and bioaccumulative nature make them a significant concern for public health, particularly in urban and industrial settings. PAHs exacerbate respiratory illnesses in COVID-19-infected individuals, are associated with a negative prognosis, and are associated with an increased risk of COVID-19-associated death [5,6,7,8,9,10,11]. PAHs can impair pulmonary immune defenses by inducing oxidative stress, disrupting epithelial barriers, and altering inflammatory signaling pathways [12,13,14,15]. Correlative human studies have shown that populations residing in areas with high ambient PAH concentrations experienced elevated COVID-19 morbidity and mortality rates, suggesting that air pollution may act as a co-factor in disease progression [5,14]. Furthermore, PAHs may exacerbate pre-existing respiratory and cardiovascular conditions such as chronic obstructive pulmonary disease (COPD) and hypertension, which are known risk factors for severe COVID-19 [4,16].

Tobacco smoke represents a major source of PAH exposure. Multiple human studies and meta-analyses have shown that current and former smokers face significantly higher risks of severe symptoms, ICU admission, hospitalization, and death [8,9,10,11], with smokers being 1.4 times more likely to develop severe COVID-19 symptoms and 2.4 times more likely to require ICU care [8]. A UK-based cohort study reported that current smokers had increased odds of hospitalization and mortality compared to never-smokers [10]. Additional retrospective analyses confirm that a history of smoking elevates the risk of severe COVID-19 outcomes [9,11]. These findings have been extended beyond tobacco cigarettes to include e-cigarettes and vapes, further expanding the groups impacted by adverse COVID-19 outcomes [17]. One PAH, benzo[a]pyrene (BP) (C_20_H_12_), has been identified as one of the major ingredients in cigarette smoke extract that influences SARS-CoV-2 infection [18]. Elevated BP exposure has been linked to increased COVID-19 disease severity and hospitalization rates, independent of both patient age and SARS-CoV-2 variant [9]. Given that approximately 28.8 million adults in the US smoke tobacco products [19], it is critical to understand how BP exposure influences the underlying pathology and progression of COVID-19 [8,20,21,22]. These findings underscore the importance of integrating environmental health perspectives into pandemic preparedness and response strategies, particularly in urban and industrialized regions where PAH exposure is prevalent.

Severe COVID-19 is characterized by systemic hyperinflammation, uncontrolled cytokine storm, and multi-organ dysfunction. Because PAHs can disrupt cellular stress response pathways required to maintain homeostasis and resolve inflammation, environmental pollutants may amplify SARS-CoV-2 pathogenesis. Understanding how environmental exposures influence these processes is critical for identifying factors that contribute to worsened disease outcomes. In this study, we aimed to use an established mouse model of SARS-CoV-2 infection to study the effects of environmental exposures on SARS-CoV-2 pathogenesis. We assessed BP exposure as a model of an environmental pollutant. Our studies describe a unique in vivo model of SARS-CoV-2 environmental exposures using transgenic k18 mice expressing the human ACE2 SARS-CoV-2 receptor (k18-hACE2). Here, we describe how BP exposure during SARS-CoV-2 infection influences lung pathology, overall survival, and immune cell recruitment in the k18-hACE2 model.

## 2. Materials and Methods

Cells, viruses, and biosafety. Ancestral SARS-CoV-2 Washington (WA) isolate (obtained from the University of Texas Medical Branch, Galveston, TX, USA) was grown, and viral titers were established using Vero E6 cells (Line CRL-1586, ATCC, Manassas, VA, USA) as previously described [23]. Viral aliquots were maintained at −80 °C until use. All work was performed in the Texas Children’s Hospital Pathogen Resource Core and Biosafety Level-3 (BSL-3) Facility in accordance with approved biosafety protocols.

Animal experiments. Six- to eight-week-old male and female k18-hACE2 mice (Strain # 034860, Jackson Laboratory, Bar Harbor, ME, USA) were used for all experiments. Mice were obtained directly from Jackson Laboratory or from a breeding colony maintained at Baylor College of Medicine, established from the Jackson Laboratory’s line. All mice were genetically confirmed prior to the studies. There were no criteria used for excluding animals in this study and no animal data points were excluded from analysis. Specific randomization methods were not used to group animals, and no external confounders were considered. Experimenters were not blinded to animal groups throughout the study, apart from histological analyses. These studies were approved by the Baylor College of Medicine Institutional Animal Care and Use Committee under protocol designation AN-8412. Individual mice were infected intranasally with 15 µL of 1 × 10^5^ plaque-forming units (PFU) of SARS-CoV-2 or phosphate-buffered saline (PBS) as a control [24]. For all infection experiments, animals were assigned a general health score based on weight loss, activity level, and breathing abnormalities. Weight and clinical disease scores were measured at least once daily throughout the experiments (Table 1). Weight change was determined as a percentage of pre-infection weight. Data normality for weight change and clinical scores was assessed by the Shapiro–Wilk test. Statistical significance for weight change was determined by multiple unpaired *t*-tests with multiple comparisons. Statistical significance for clinical scores was determined by the Wilcoxon matched pairs sign ranked test and Mann–Whitney test with multiple comparisons. *p*-value ≤ 0.05 denotes statistical significance.

Environmental exposure. To assess changes in SARS-CoV-2 pathogenesis due to environmental exposure to pollutants, mice were treated with 20 mg/kg of BP intranasally at a dose of 1uL/gram of mouse weight once prior to SARS-CoV-2 infection (n = 10/group). This dose was selected based on previous murine studies demonstrating that single pulmonary BP exposure induce significant molecular and pathological changes in the lung, providing a biologically relevant model of pollutant exposure while minimizing repeated handling and dosing-related stress [24]. The 20 mg/kg dose of BP represents an environmentally relevant BP exposure level and was selected based on its environmental relevance to human exposure near Superfund sites [25], exposure from dietary intake [26,27], and allometric scaling and physiologically based pharmacokinetic modeling considerations [28,29]. Such exposures have been shown to exacerbate clinical disease in mice, suggesting potential applicability to human health outcomes. This aligns with findings from human studies, supporting the dose’s relevance and translational value. Corn oil (CO) was used as a vehicle control for PAH exposure. At 5 days post-infection (DPI), lungs were collected to assess viral load, histopathology, and immune cell populations.

Survival experiments. In separate experiments to assess changes in survival and disease state, mice were treated with BP or CO (n = 24/group) prior to infection with SARS-CoV-2 as described above. The primary outcome used to determine sample size was survival. Sample size was determined based on detecting a 25% difference in survival with 80% power and α = 0.05. Sex-specific effects were not accounted for, and the study was not powered to assess sex-specific differences. Weight change and disease scores were measured up to 11 DPI, or until humane euthanasia criteria were met. Viral load quantification and histological staining were performed on lungs. Statistical analysis was performed using GraphPad Prism (version 10). Statistical significance was determined by the Log-rank (Mantel–Cox) test to assess differences in survival between the two groups over time. *p*-value ≤ 0.05 denotes statistical significance.

Lung viral load. To determine lung viral load, harvested lungs were weighed and combined with 500 µL of complete Dulbecco’s Modified Eagle Medium (DMEM) containing 5% fetal bovine serum (FBS), high glucose, L-glutamaine, sodium pyruvate, 1× non-essential amino acids, and 1× penicillin-streptomycin. Lungs were homogenized at 25 Hz for 5 min using the TissueLyser II (QIAGEN, Hilden, Germany). Homogenates were used for a standard plaque assay as previously described [23]. Briefly, lung homogenates were serially diluted in complete DMEM from 10-1 to 10-4 and used to infect Vero E6 cells for 1 h at 37 °C. After infection, cells were overlaid with a 1:1:1 mixture of complete DMEM, 2× Modified Eagle Medium (MEM), and 1.5% agarose diluted in water, and incubated for 48 h at 37 °C. Cells were fixed with 10% neutral buffered formalin for 30 min, overlay plugs were removed, and cells were stained with 0.5% crystal violet prior to counting plaques. Statistical analysis was performed using GraphPad Prism (version 10). Normality was assessed by the Shapiro–Wilk test and statistical significance was determined by either the Mann–Whitney test or the Kruskal–Wallis test with multiple comparisons, depending on the normal distribution of the data. *p*-value ≤ 0.05 denotes statistical significance.

Lung Histology. Lungs were inflated with 10% neutral buffered formalin after euthanasia. Inflated lungs were fixed in additional formalin over the course of 72 h before removal from the BSL-3 facility. Pathology and histology services were provided by the MD Anderson Veterinary Pathology Services laboratory in the Department of Veterinary Medicine and Surgery. Lungs were embedded in paraffin, sectioned, and stained with hematoxylin and eosin prior to imaging on an Aperio AT2 (Leica, Wetzlar, Germany). Gross histopathological images of entire lungs are included in the Supplemental Materials (Figures S2–S4). Images were scored for cellular infiltrates and inflammation by a blinded co-investigator using the acute lung injury (ALI) scoring system as previously described [30,31,32]. Briefly, histological images were analyzed using FIJI/ImageJ (version x86-64). Lung tissue regions of interest were traced, and randomized fields of view (FOV) were generated using a macro for analysis (Figure S1). FOVs were scored based on the following parameters: polymorphonuclear neutrophils (PMN) in the alveolar space, PMN in the interstitial space, hyaline membranes, proteinaceous debris in airspaces, and alveolar septal thickening. Total ALI scores were determined from 20 FOVs, and statistical analyses were performed using one-way ANOVA with multiple comparisons. *p*-value ≤ 0.05 denotes statistical significance. Histopathology scores are represented as the mean ± standard error of the mean (SEM).

Flow cytometry on immune cell populations. Mouse lungs were digested with collagenase D and DNase I and then mechanically dissociated through 70 µm strainers into single-cell suspensions for flow cytometry (Table 2) (n = 6/group). RBC lysis was performed using 1× RBC Lysis Buffer (BioLegend, San Diego, CA, USA) according to the manufacturer’s instructions prior to staining with fluorophore-conjugated monoclonal antibodies against innate and adaptive immune cell markers (Table 3). All data were acquired on the Attune NxT cytometer (Thermo Fisher, Waltham, MA, USA) with an autosampler and analyzed with FlowJo software (version 9). The gating strategy for quantifying cell populations is included in the Supplemental Materials (Figure S5). Statistical analysis was performed using GraphPad Prism (version 10). Normality was assessed by the Shapiro–Wilk test, and statistical significance was determined by one-way ANOVA with multiple comparisons. *p*-value ≤ 0.05 denotes statistical significance.

## 3. Results

### 3.1. BP Exposure Decreases Survival in a Mouse Model of SARS-CoV-2 Infection

**Figure 1:**
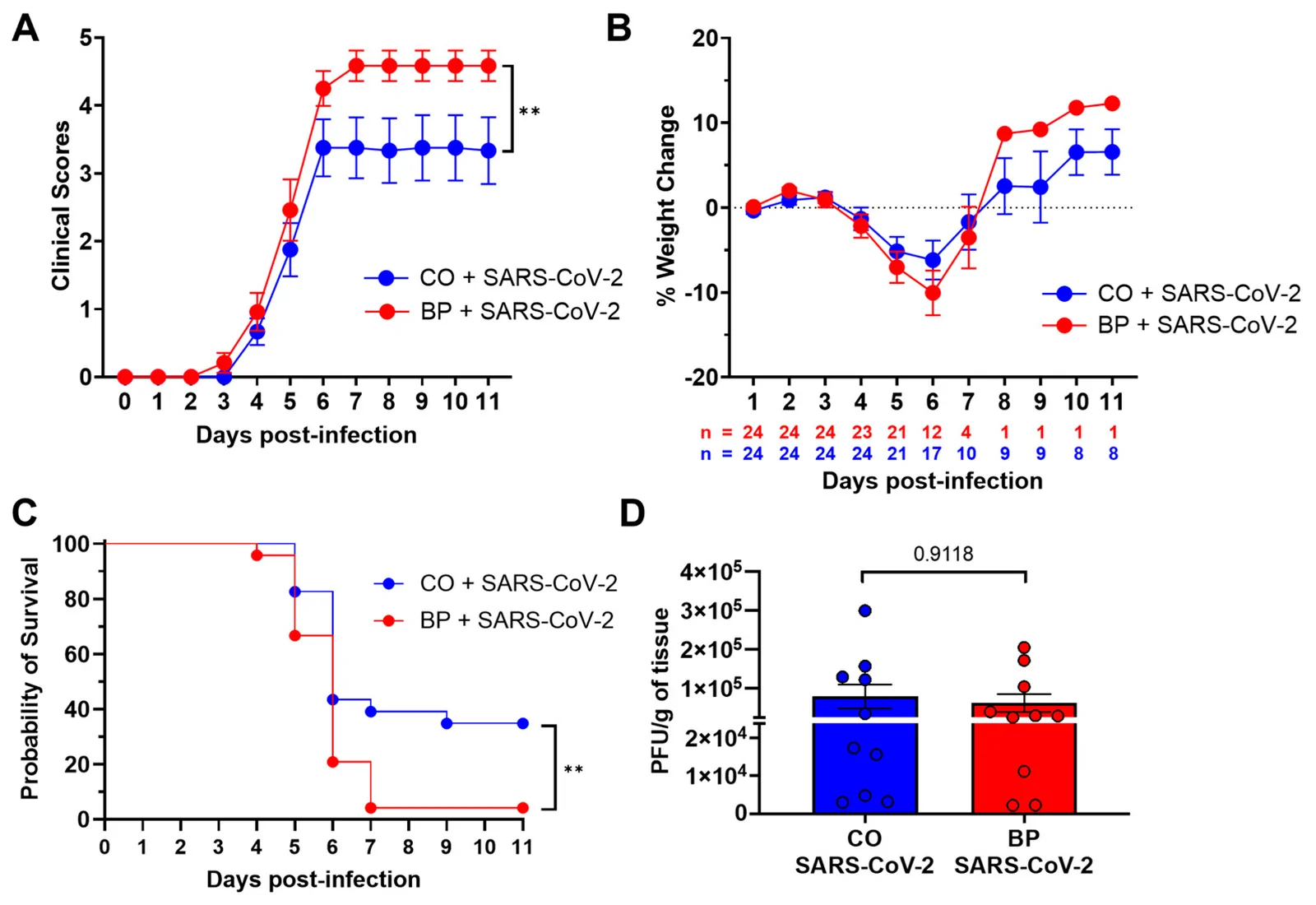
**BP exposure worsens clinical outcomes during SARS-CoV-2 infection.** K18-hACE2 mice (n = 24/group) were infected with the SARS-CoV-2 WA isolate after treatment with or without BP. Mice were monitored for clinical disease scores (**A**), weight change (**B**), and overall survival (**C**) up to 11 DPI. Number of remaining animals per group at each time point is noted in panel B. Viral load (**D**) was assessed on lung homogenates on 5 DPI (n = 10/group). Normality was assessed by Shapiro–Wilk test and statistical significance was determined by Wilcoxon matched pairs sign ranked test (**A**), multiple unpaired *t*-tests (**B**), Log-rank (Mantel–Cox) test (**C**), or Mann–Whitney test (**D**). Data are presented as means and error bars represent ±SEM. *p*-value ≤ 0.05 denotes statistical significance (** = *p* ≤ 0.01).

To assess how PAHs influenced SARS-CoV-2 pathogenesis in mice, BP or CO vehicle control was intranasally administered to k18-hACE2 mice prior to infection with the SARS-CoV-2 WA strain or a mock infection with PBS. Clinical outcomes were monitored post-infection to assess disease severity. Mice exposed to BP exhibited significantly greater clinical disease scores compared to mice that received vehicle treatment, indicating exacerbated disease progression (Figure 1A). Similarly, mice treated with BP experienced more pronounced weight loss over the course of infection (Figure 1B) and had significantly reduced survival rates compared with vehicle-treated controls (*p* = 0.0093) (Figure 1C). Despite marked differences in clinical disease scores and survival probability with BP exposure during SARS-CoV-2 infection, there were no significant differences in lung viral load at 5 DPI between CO- or BP-treated groups (Figure 1D). These data suggest that the worsened disease phenotype associated with BP exposure is not due to respiratory viral burden but may reflect altered host responses or localized effects in the respiratory tract.

### 3.2. BP Exposure May Exacerbate Lung Pathology During SARS-CoV-2 Infection

**Figure 2:**
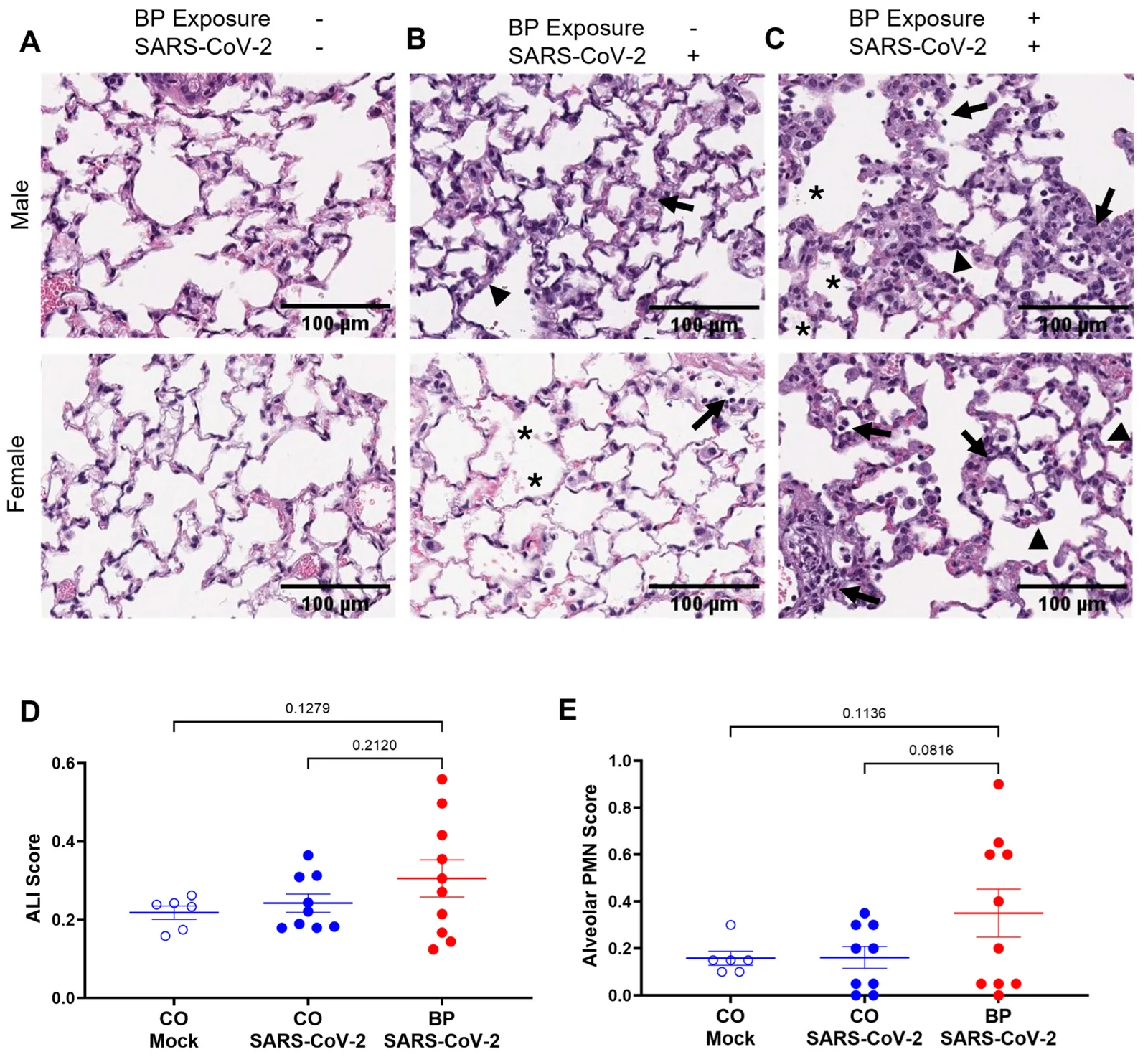
**BP exposure may exacerbate lung histopathology during SARS-CoV-2 infection.** K18-hACE2 mice were infected with SARS-CoV-2 WA isolate after treatment with or without BP. Lungs were inflated in formalin and embedded, sectioned, and H&E stained for histopathology at 5 DPI (n = 10/group) ((**A**–**C**); top panel males, bottom panel females). H&E images from lungs harvested between 4 and 6 DPI from combined studies (n = 6–10/group) were scored for overall acute lung injury (**D**) by evaluating 5 parameters: neutrophils in alveolar space, neutrophils in interstitial space, hyaline membranes, proteinaceous debris, and alveolar septal thickening. Alveolar neutrophil scores were averaged across each condition (**E**). Proteinaceous debris is represented by asterisks, neutrophil infiltration is represented by arrows, and septal thickening is represented by arrowheads (**A**–**C**). Histopathology ALI scores and alveolar neutrophil scores are represented as mean and error bars as ±SEM. Normality was assessed by Shapiro–Wilk test and statistical significance was determined by one-way ANOVA with multiple comparisons.

To evaluate the impact of PAH exposure on lung histopathology during SARS-CoV-2 infection, k18-hACE2 mouse lungs were harvested between 4 and 6 DPI as a representative time frame of severe disease. Lungs were inflated in formalin, embedded, and sectioned for histological staining and analysis. Mice exposed to BP during SARS-CoV-2 infection exhibited more severe pulmonary pathology including alveolar collapse and neutrophilic infiltration, as observed by histopathology of representative lung sections (Figure 2A–C). To quantify pathological differences, ALI scoring was performed by blinded reviewers according to established criteria. Twenty random FOVs were generated per lung section and scored based on the presence of alveolar PMNs, interstitial PMNs, septal thickening, proteinaceous debris, and hyaline membrane formation [30,32]. BP-exposed mice had higher ALI histopathology scores that trended upward during SARS-CoV-2 infection compared to those that received CO vehicle control or underwent mock infection, although the differences were not statistically significant. (Figure 2D). Additionally, alveolar PMN counts were averaged across all FOVs to generate a weighted alveolar PMN score for each animal. Mice treated with BP prior to SARS-CoV-2 infection had trending increases in alveolar PMN scores in the lungs compared to mock-treated and mock-infected animals (Figure 2E). Although these differences were not statistically significant, there was a marked trend toward higher disease severity in the lungs as presented by ALI scoring and PMN infiltration. Collectively, these findings suggest that prior exposure to BP may exacerbate SARS-CoV-2-induced pulmonary pathology, likely through enhanced inflammatory responses and disruption of alveolar integrity.

### 3.3. BP Exposure Does Not Alter Lung Innate and Adaptive Immune Cell Infiltration During SARS-CoV-2 Infection

**Figure 3:**
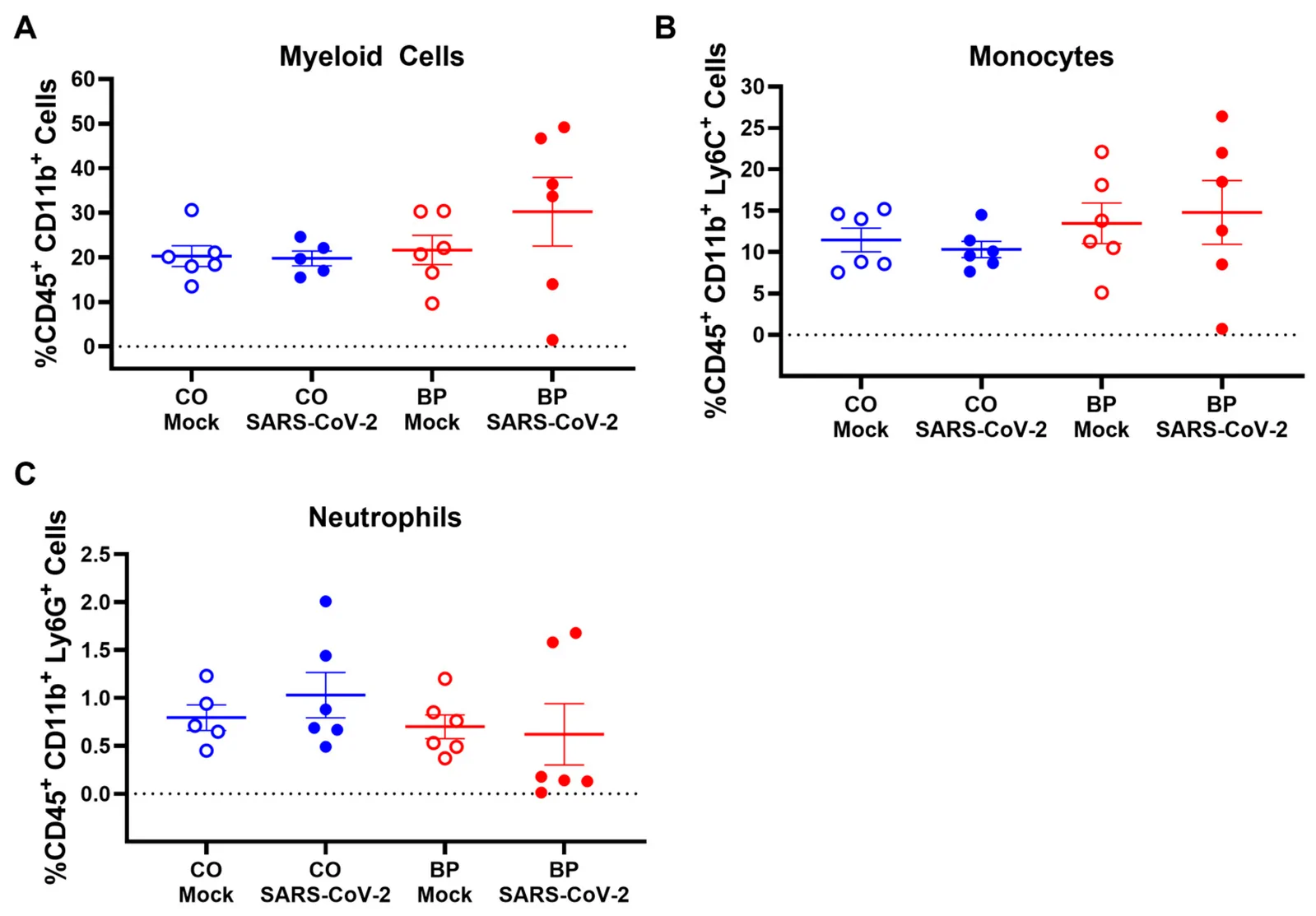
**Exposure to BP during SARS-CoV-2 infection has limited effects on innate immune cell populations in mouse lungs.** K18-hACE2 mice were infected with the SARS-CoV-2 WA isolate after treatment with or without BP. Lungs were homogenized into single-cell suspensions at 5 DPI and stained for flow cytometry (n = 6/group). Populations of myeloid cells (**A**), monocytes (**B**), and neutrophils (**C**) were quantified. Data are presented as means and error bars represent ±SEM. Normality was assessed by Shapiro–Wilk test and statistical significance was determined by one-way ANOVA with multiple comparisons.

To better understand if environmental exposures impacted immune cell recruitment during SARS-CoV-2 infection, BP was administered to k18-hACE2 mice prior to SARS-CoV-2 infection. Lung tissues were harvested at 5 DPI, and single-cell suspensions were analyzed by flow cytometry to characterize immune cell populations. Multiparameter flow cytometry panels were used to quantify both innate and adaptive immune subsets (Table 2). BP exposure caused a trending decrease in lung myeloid cells during SARS-CoV-2 infection compared to mock-infected controls, although these differences were not statistically significant (Figure 3A). There were also no changes in monocyte populations in k18-hACE2 mouse lungs when exposed to BP during infection compared to infected mice exposed to CO (Figure 3B). Although not statistically significant, BP exposure appeared to result in a trending decrease in neutrophil levels relative to CO-treated, infected mice (Figure 3C).

**Figure 4:**
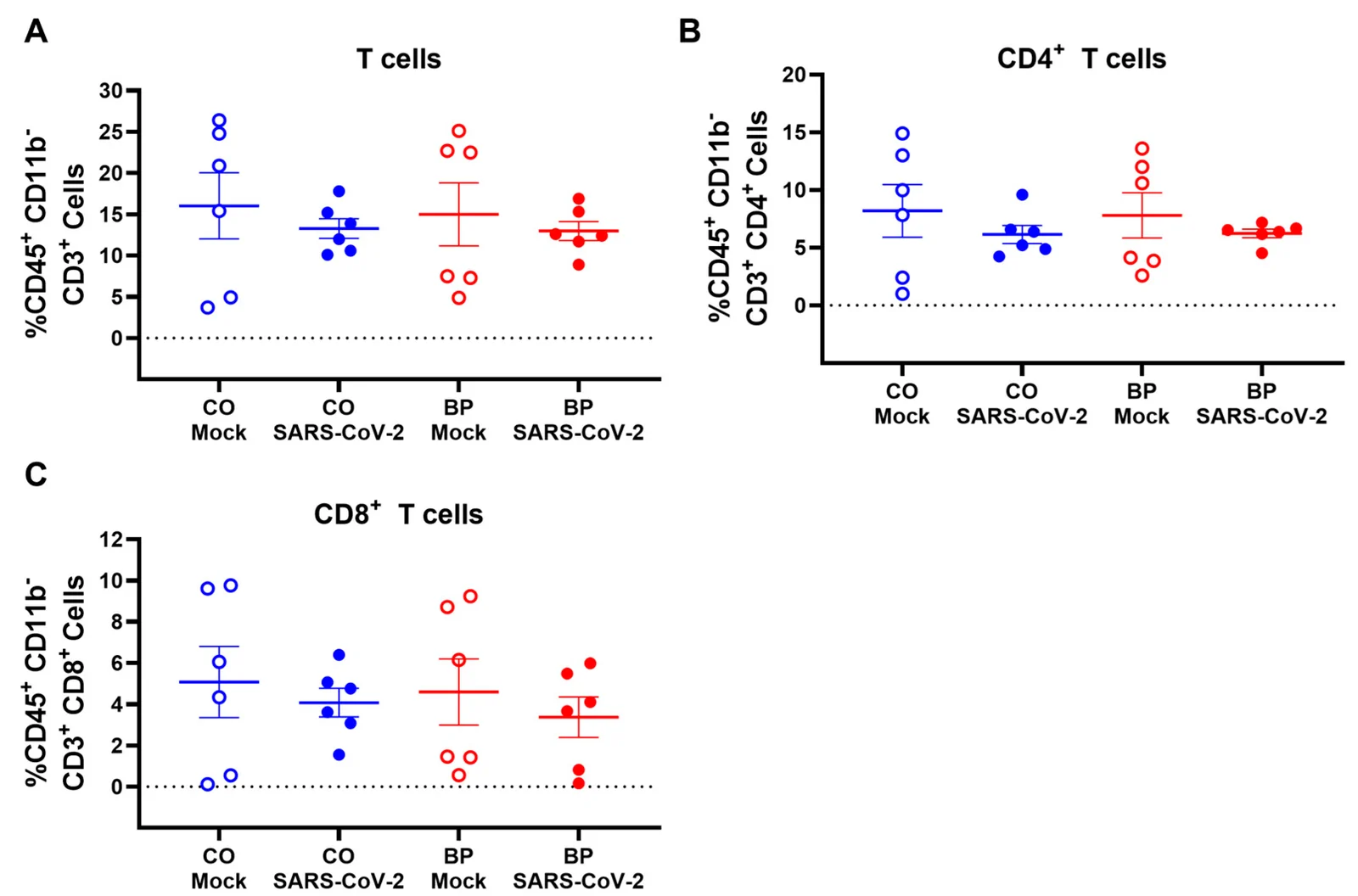
**Exposure to BP during SARS-CoV-2 infection does not alter adaptive immune cell populations in mouse lungs.** K18-hACE2 mice were infected with the SARS-CoV-2 WA isolate after treatment with or without BP. Lungs were homogenized into single-cell suspensions at 5 DPI and stained for flow cytometry (n = 6/group). Populations of CD3+ T cells (**A**), CD4+ T cells (**B**), and CD8+ T cells (**C**) were quantified. Data are presented as means and error bars represent ±SEM. Normality was assessed by Shapiro–Wilk test and statistical significance was determined by one-way ANOVA with multiple comparisons.

Adaptive immune cell populations were also quantified in mouse splenocytes by flow cytometry. K18-hACE2 mouse lungs had no changes in total CD3+ T cell populations between SARS-CoV-2-infected and mock-infected groups independently of PAH exposure (Figure 4A). Additionally, both CD4+ and CD8+ T cell populations were similar in the lungs of SARS-CoV-2-infected mice regardless of environmental exposure (Figure 4B,C). Overall, there were no significant differences in adaptive immune cell populations between CO- and BP-treated groups during SARS-CoV-2 infection (Figure 4). Taken together, these data indicate that BP exposure may have a limited impact on innate immune cell populations, although no significant differences were observed. Additionally, BP exposure does not significantly alter adaptive immune cell populations during SARS-CoV-2 infection at 5 DPI.

## 4. Discussion

In this study, we tested the hypothesis that exposure to BP exacerbates the severity of SARS-CoV-2 infection. We demonstrated that BP administration significantly reduces overall survival in SARS-CoV-2-infected animals. Our data also suggest that BP exposure may increase lung pathology in early infection. These findings also demonstrate that BP exposure does not alter innate and adaptive immune cell recruitment to the lungs during SARS-CoV-2 infection. Together, these data support the hypothesis that PAH exposure exacerbates SAR-CoV-2 clinical outcomes, although there are limitations to this study and further investigation is needed.

Our data suggest that BP exposure does not alter outcomes during early SARS-CoV-2 infection but decreases the probability of survival (Figure 1) and suggest a trend toward worsening lung pathology in mice (Figure 2). However, there have been conflicting results with regard to how BP can ameliorate or exacerbate COVID-19-associated pathology. One study demonstrates that BP downregulates the SARS-CoV-2 angiotensin-converting enzyme 2 (ACE2) receptor by ubiquitination mechanisms, thereby reducing SARS-CoV-2 pseudovirion entry into host cells and reducing infection [18]. Conversely, another study highlighted BP as facilitating SARS-CoV-2 infection by upregulating both ACE2 and transmembrane protease serine 2 (TMPRSS2) through nuclear receptor upregulation and promoter interaction, as well as increasing pseudovirus susceptibility [33]. In patients, BP exposure caused increased early COVID-19 symptoms such as fever, fatigue, and hyperinflammation, and overall worse clinical outcomes requiring hospitalization regardless of age or SARS-CoV-2 variant [14,34]. Additional research is needed to reconcile these differences in findings and elucidate how BP and environmental exposures influence SARS-CoV-2 pathogenesis. Future studies will measure viral titers over time and assess changes in other affected organs such as the brain to elucidate the proximal cause of death.

In addition to these findings, we characterized changes in immune cell populations associated with BP exposure during SARS-CoV-2 infection. We demonstrated that BP exposure does not significantly alter innate immune cell populations during SARS-CoV-2 infection, with no differences in myeloid, monocyte, or neutrophil populations (Figure 3). These data are not consistent with previous literature suggesting that dysregulation of myeloid cells contributes to the pathogenesis of severe COVID-19, including the development of acute respiratory distress syndrome (ARDS), cytokine storm, and lymphopenia [35]. Additionally, we observed discrepancies in neutrophil infiltration in these studies. While histopathological analysis revealed a trend toward increased neutrophil presence, flow cytometry did not detect statistically significant changes, likely due to limited sample size or variability in cell recovery. Additional studies are needed to quantify additional innate immune populations. Myeloid lineages also include populations of both macrophages and dendritic cells which could be contributing to pathological differences. Furthermore, natural killer (NK) cells are also documented to be important in SARS-CoV-2 infection. Current hypotheses and clinical reports describe depleted circulating NK cells in the peripheral blood of COVID-19 patients due to their recruitment to the lungs [36,37]. Future studies assessing circulating immune cells and incorporating additional time points could shed light on these differences.

In assessing adaptive immune cell populations, we observed no significant differences in general CD3+, CD4+, and CD8+ T cells in the lungs during infection (Figure 4). These data were inconsistent with reports of T cell lymphopenia in patients with severe COVID-19 [38,39], as well as with observations of normal or increased T cell counts in individuals with mild disease presentation [38,40]. One caveat of this study is that 5 DPI may be too early to accurately quantify changes in adaptive immune populations during infection. Future studies will assess changes in lung immune cell populations at later time points that would more accurately represent adaptive responses and capture potential immune dysregulation during SARS-CoV-2 infection. Changes in lung immune cells could contribute to excess cytokine production and inflammatory pathology during SARS-CoV-2 infection, potentially influencing disease severity and outcomes. Additional studies will also quantify inflammatory cytokines and chemokines that may contribute to these changes.

As tobacco smoke is known to influence immune and inflammatory processes and increase SARS-CoV-2 pathogenesis [41], smoking and subsequent PAH exposure highlight important considerations for overall health outcomes after acute COVID-19 infection. Furthermore, PAHs have been shown to activate the aryl hydrocarbon receptor (AhR), a transcription factor involved in xenobiotic metabolism and immune regulation [42]. AhR activation by PAHs can drive oxidative stress through the induction of CYP1A1/CYP1B1-mediated reactive oxygen species (ROS) production. Elevated ROS levels contribute to epithelial injury and barrier dysfunction, promote vascular endothelial damage and permeability, and stimulate the release of pro-inflammatory cytokines and chemokines. AhR-dependent pathways amplify local inflammation and tissue remodeling, contributing to disease pathogenesis across multiple organ systems. AhR activation by PAHs may interfere with antiviral immune responses, including interferon signaling, thereby compromising host defense against SARS-CoV-2. A limitation of this study is that AhR activation and cytokine production were not directly assessed. Future studies are needed to assess mechanistic changes in host immune responses and host pathology due to PAH-AhR signaling during SARS-CoV-2 infection, and how smoking could influence these changes, as well as effects on other organ systems.

Overall, these findings demonstrate that exposure to BP as a representative PAH reduces overall survival and may worsen lung pathology during SARS-CoV-2 infection in K18-hACE2 mice. Our study highlights the complex interplay between environmental exposure and viral infections. These findings underscore the need for integrated environmental and infectious disease surveillance, as well as targeted public health interventions to mitigate the compounded risks posed by environmental pollutants and emerging pathogens like SARS-CoV-2. Further studies are needed to elucidate the underlying mechanisms driving these differences, assess dose-dependent effects, and determine the relevance of these findings in human populations with chronic PAH exposure.

## Figures and Tables

**Table 1:** Clinical disease scoring system applied to SARS-CoV-2-infected mice throughout these studies.

Score	Description
0	Healthy, no signs of disease. Body tone appropriate. Weight is consistent or increasing from the previous day. Normal activity level.
1	~5% weight loss and/or no signs of disease or very mild signs of disease. No hunching. Normal to slightly decreased activity level.
2	5–10% weight loss and/or mild signs of disease. Decreased activity levels.
3	10–15% weight loss and/or onset of breathing abnormalities (rapid shallow breaths), some hunching, mildly lethargic.
4	15–20% weight loss and/or rapid breathing abnormalities, hunching, lethargic.
5	>20% weight loss, respiratory distress or moribund condition. Immediate euthanasia required.

**Table 2:** Lung immune cell populations distinguished by flow cytometry.

Cell Population	Markers
Myeloid Lineage	CD45+, CD11b+
Monocytes	CD45+, CD11b+, Ly-6C+
Neutrophils	CD45+, CD11b+, Ly-6G+
T cells	CD45+, CD11b−, CD3+
CD4+ T cells	CD45+, CD11b−, CD3+, CD4+
CD8+ T cells	CD45+, CD11b−, CD3+, CD8+

**Table 3:** Immune cell marker antibodies used for flow cytometry.

Markers	Fluorophore	Clone	Manufacturer
CD45	APC-eFluor780	30-F11	Thermo Fisher (Waltham, USA)
CD11b	AF594	M1/70	BioLegend (San Diego, USA)
Ly-6C	AF488	HK1.4	Thermo Fisher (Waltham, USA)
Ly-6G	PerCP-eFluor710	1A8-Ly6g	Thermo Fisher (Waltham, USA)
CD3	PE-Cy7	145-2C11	Thermo Fisher (Waltham, USA)
CD4	AF647	GK1.5	BioLegend (San Diego, USA)
CD8a	Pacific Blue	5H10	Thermo Fisher (Waltham, USA)
Live/Dead Fix Aqua			Thermo Fisher (Waltham, USA)

## Data Availability

The raw data supporting the conclusions of this article will be made available by the authors on request.

## Supplementary Materials

The following supporting information can be downloaded at: https://www.mdpi.com/article/10.3390/v18080823/s1, Figure S1: FIJI/ImageJ macro for ALI score analysis; Figure S2: Histological staining on whole mouse lungs without BP exposure or SARS-CoV-2 infection; Figure S3: Histological staining on whole mouse lungs without BP exposure during SARS-CoV-2 infection; Figure S4: Histological staining on whole mouse lungs after BP exposure during SARS-CoV-2 infection; Figure S5: Flow cytometry gating scheme.
