# Supplementary material for: The Environmental Polycyclic Aromatic Hydrocarbon (PAH) Benzo[a]pyrene (BP) Alters SARS-CoV-2 Pathogenesis in a Mouse Model of Disease
Source: Viruses. 2026 Jul 26;18(8):823. doi: 10.3390/v18080823 (PMC13517826; doi:10.3390/v18080823)
Supplement: Supplementary file 1 [file viruses-18-00823-s001.zip › 07242026_Updated Supplemental Figs.pdf]

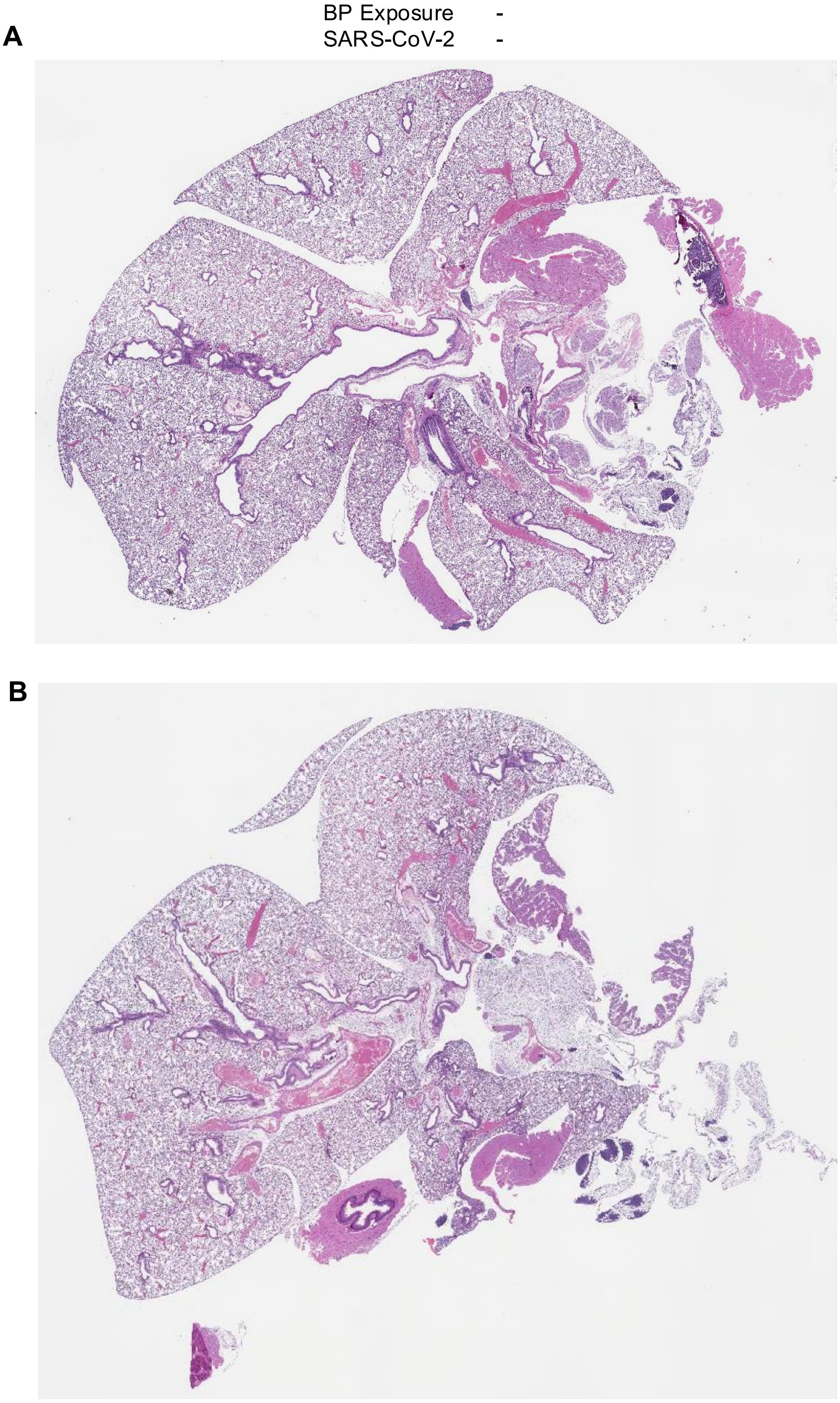

Fig S2. Histological staining on whole mouse lungs without BP exposure or SARS-CoV-2 infection. Male (**A**) and female (**B**) K18-hACE-2 mice were mock infected with PBS after treatment with CO. Lungs were inflated in formalin and embedded, sectioned, and H&E stained for histopathology at 5 DPI.

BP Exposure -
SARS-CoV-2 +

A

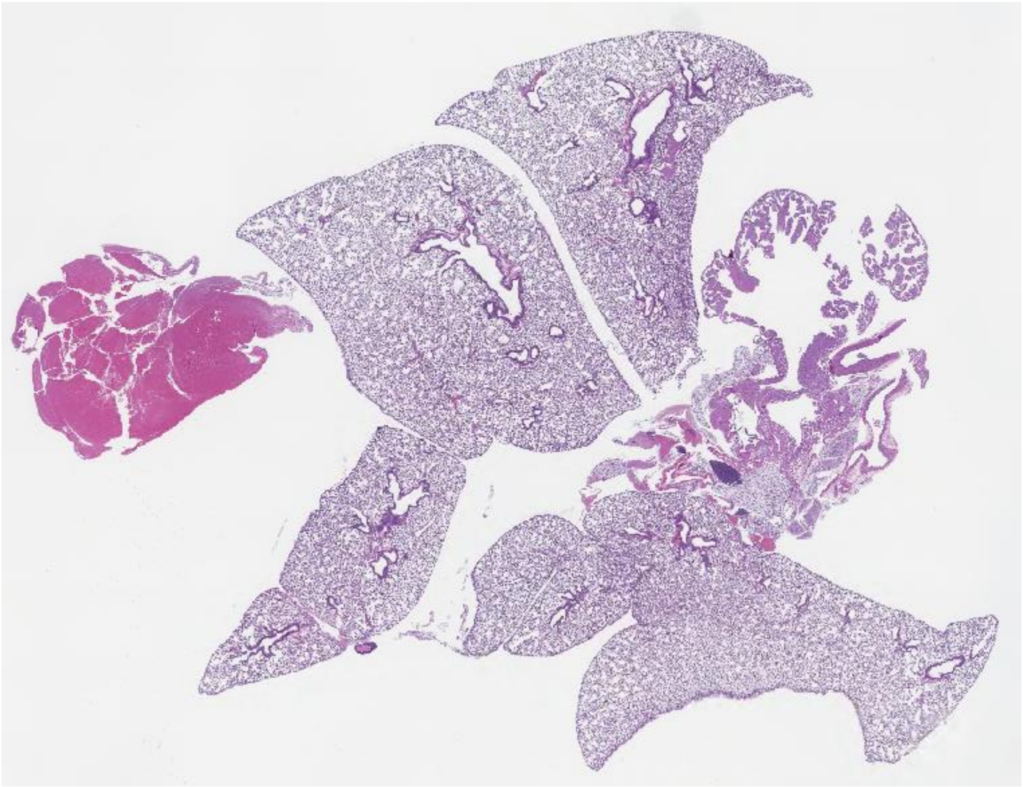

B

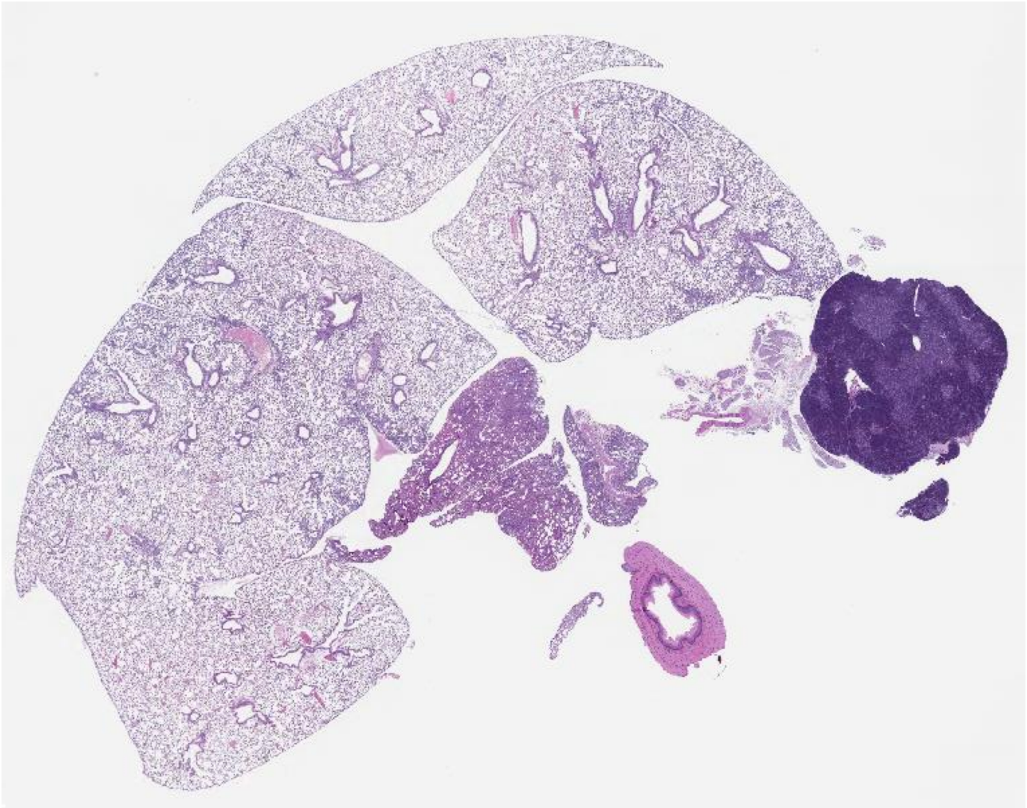

Fig S3. Histological staining on whole mouse lungs without BP exposure during SARS-CoV-2 infection. Male (**A**) and female (**B**) K18-hACE-2 mice were infected with SARS-CoV-2 WA strain after treatment with CO. Lungs were inflated in formalin and embedded, sectioned, and H&E stained for histopathology at 5 DPI.

BP Exposure +
SARS-CoV-2 +

A

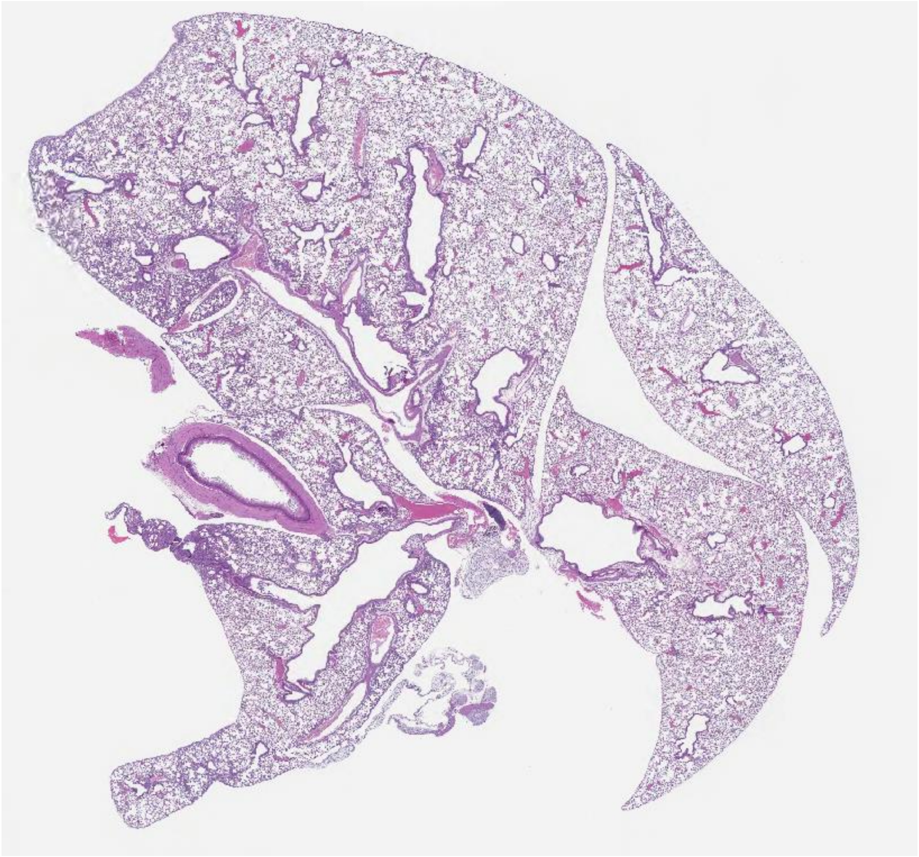

B

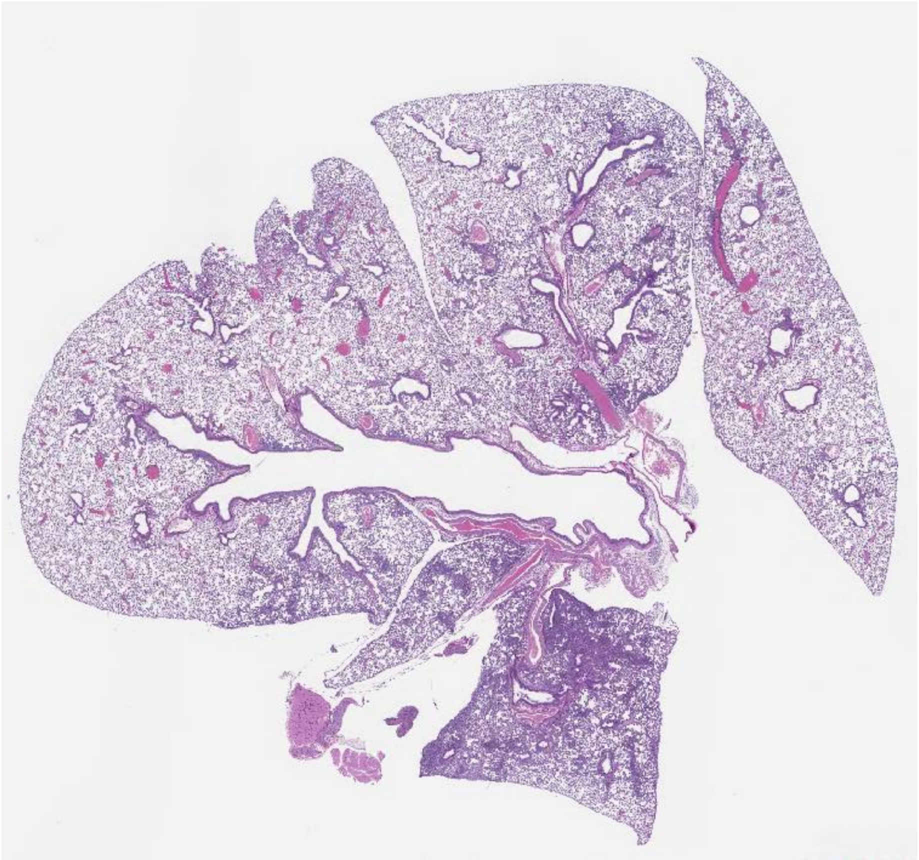

Fig S4. Histological staining on whole mouse lungs after BP exposure during SARS-CoV-2 infection. Male (**A**) and female (**B**) K18-hACE-2 mice were infected with SARS-CoV-2 WA strain after treatment with BP. Lungs were inflated in formalin and embedded, sectioned, and H&E stained for histopathology at 5 DPI.

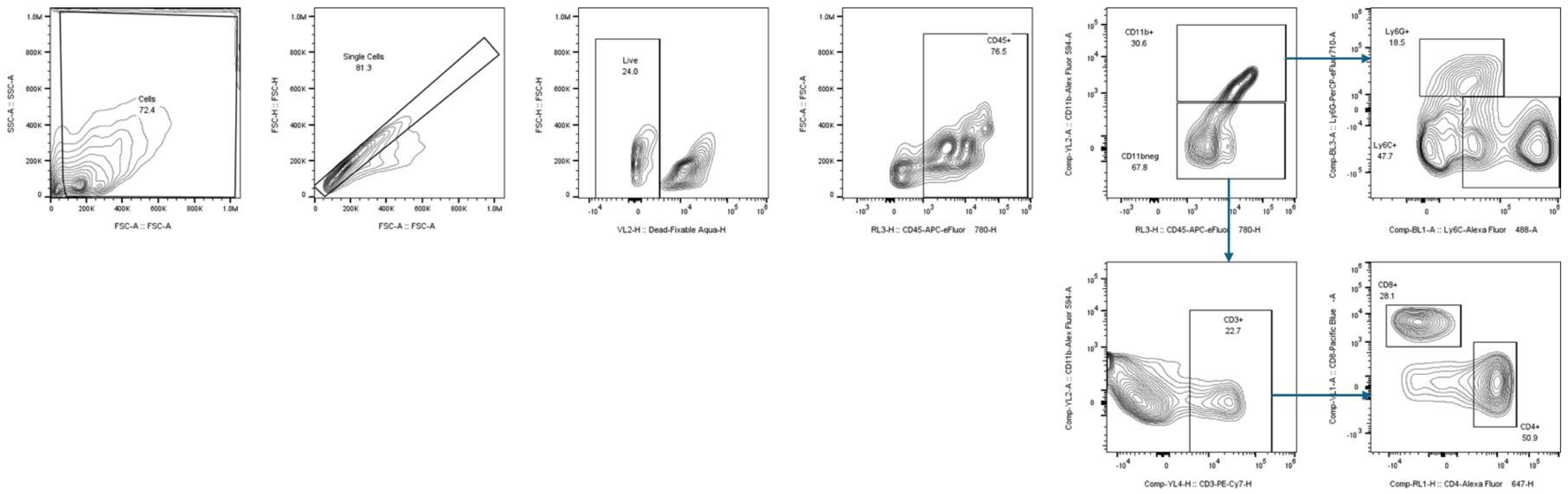

Fig S5. Flow cytometry gating scheme. Immune cell populations were identified by flow cytometry. Populations were separated according to the markers and gates indicated here.
