# Supplementary material for: The Environmental Polycyclic Aromatic Hydrocarbon (PAH) Benzo[a]pyrene (BP) Alters SARS-CoV-2 Pathogenesis in a Mouse Model of Disease
Source: Viruses. 2026 Jul 26;18(8):823. doi: 10.3390/v18080823 (PMC13517826; doi:10.3390/v18080823)
Supplement: Supplementary file 1 [file viruses-18-00823-s001.zip › Figure S1.pdf]

Supplemental Material: Fiji/ImageJ Macro

```
waitForUser("Halt!", "Make a selection(s) and then proceed by clicking [OK].\nYou can have
MULTIPLE selections by holding down [Shift] when making a new selection.\nYou can EXCLUDE areas
by holding down [Alt] when selecting.\nTip: You can make a large selection with the [Freehand
Selection Tool] then remove areas using [Alt]-[Selection Brush Tool].");
SEL=selectionType();
if (SEL>-1) {
        original=getTitle();
        output = getDirectory("Where should the processed files go?");
        run("Duplicate...", "title=Area");
        selectWindow("Area");
        run("Line Width...", "line=10");
        run("Draw");
        saveAs("Jpeg", output + original + "-Area.jpg");
        close();
        run("Select None");
        saveSettings();
        setForegroundColor(255,0,0);
        w = getNumber("How wide (in pixels) is the box", 640);
        h = getNumber("How high (in pixels) is the box", 480);
        width = getWidth()-w;   // width of the randomly placed ROI
        height = getHeight()-h; // height of the randomly placed ROI
        RoisN = getNumber("How many ROIs should be made?", 20); // number of ROIs
        Runs = getNumber("How many attempts should be made? (The higher the number, the longer it
will take)", 50); //Runs
        trials=RoisN*Runs; //maximum trials to avoid infinite loop (origianally 1000)

        i=0;
        j=0;
        xa=newArray(RoisN);
        ya=newArray(RoisN);

        run("Duplicate...", "title=Reference");
        selectWindow("Reference");
        run("8-bit"); //makes it greyscale
        run("RGB Color"); //RGB to display colours
        run("Restore Selection");
        run("Make Inverse");
        run("Fill");
        run("Select None");

        while (i<RoisN && j<trials){
        x = random()*width;
        y = random()*height;
        j++;
         //Check for pixels with value (255,0,0):
         flag= -1;
         makeRectangle(x, y, w, h);
         //Scanning the rectangle perimeter should be faster than scanning the whole box.
         //This is slower, as checks all the points in the box:
         for (xs=x;xs<x+w;xs++){
             for (ys=y;ys<y+h;ys++){
                 if (getPixel(xs,ys)==-65536) // pixel is (255,0,0)
```

```
                flag=0;
            }
        }
        if (flag==-1){
            xa[i]=x;
            ya[i]=y;
            run("Fill");
            i++;
        }
        }

        close();
        run("ROI Manager..."); //open ROI manager
        selectWindow(original);
        setForegroundColor(255,255,0);
        run("Set Measurements...", "centroid bounding display add redirect=None decimal=0"); //to
ensure measurements are correct.
        for (j=0;j<i;j++){
        makeRectangle(xa[j], ya[j], w, h);
        roiManager("Add");
        run("Duplicate...", original + "-" + j+1 + ".tif");
        saveAs("Jpeg", output + original + "-" + j+1 + "-ROI.jpg");
        close();
    }
        roiManager("deselect");
        roiManager("measure");
        roiManager("Save",output + original + "-ROI.zip");
        saveAs("Results",output + original + "-RESULTS.csv");
        roiManager("show all with labels");
        run("Line Width...", "line=5");
        roiManager("Show All");
        roiManager("Draw");
        saveAs("Jpeg", output + original + "-MAP.jpg");
        restoreSettings();
        run("Select None");
        run("Close All");
        selectWindow("Results");
        run("Close");
        selectWindow("ROI Manager");
        run("Close");
        showMessage("All Done! Have a nice day :)");
}
else {
        showMessage("No selection was made");
}
```
